# Basic vaginal pH, bacterial vaginosis and aerobic vaginitis: prevalence in early pregnancy and risk of spontaneous preterm delivery, a prospective study in a low socioeconomic and multiethnic South American population

**DOI:** 10.1186/1471-2393-14-107

**Published:** 2014-03-19

**Authors:** Leticia Krauss-Silva, Antonio Almada-Horta, Mariane B Alves, Karla G Camacho, Maria Elizabeth L Moreira, Alcione Braga

**Affiliations:** 1 Health Technology Assessment Unit, National School of Public Health, Oswaldo Cruz Foundation, Brazilian Health Ministry, R Leopoldo Bulhões, 1480, room 714, Manguinhos, Rio de Janeiro 21041-210, Brazil; 2 Federal University of Rio de Janeiro Medical School, Av. Brigadeiro Trompowski, Rio de Janeiro 21044-020, Brazil; 3 Institute of Mathematics, Federal University of Rio de Janeiro, Av. Athos da Silveira Ramos - 149, Rio de Janeiro 21941-909, Brazil; 4 Clinical Research Unit, Fernandes Figueira Institute, Oswaldo Cruz Foundation, Brazilian Health Ministry, Av. Rui Barbosa, 716, Rio de Janeiro 22250-020, Brazil; 5 PROCEP, Pró-Cardíaco, R. General Polidoro 142, Rio de Janeiro 22280-003, Brazil

**Keywords:** Bacterial vaginosis, Spontaneous preterm delivery, Aerobic vaginitis, Vaginal pH level, Prevalence, Relative risk, Ethnicity

## Abstract

**Background:**

Bacterial vaginosis (BV) increases the risk of spontaneous preterm deliveries (PD) in developed countries. Its prevalence varies with ethnicity, socioeconomic conditions and gestational age. Aerobic vaginitis (AV) has also been implicated with spontaneous PD. The present study aimed to estimate the prevalence of asymptomatic BV, the accuracy of vaginal pH level to predict BV and to estimate the risk of spontaneous PD <34 and <37 weeks’ gestation of BV and AV.

**Methods:**

Women attending prenatal public services in Rio de Janeiro were screened to select asymptomatic pregnant women, < 20 weeks’ gestation, with no indication for elective PD and without risk factors of spontaneous PD. Vaginal smears of women with vaginal pH > = 4.5 were collected to determine the Nugent score; a sample of those smears was also classified according to a modified Donders’ score. Primary outcomes were spontaneous PD < 34 and <37 weeks’ gestation and abortion.

**Results:**

Prevalence of asymptomatic BV was estimated in 28.1% (n = 1699); 42.4% of the smears were collected before 14 weeks’ gestation. After an 8-week follow up, nearly 40% of the initially BV positive women became BV negative. The prevalence of BV among white and black women was 28.1% (95% CI: 24.6%-32.0%) and 32.5% (95% CI: 28.2%-37.2%), respectively. The sensitivity of vaginal pH= > 4.5 and = > 5.0 to predict BV status was 100% and 82%, correspondingly; the 5.0 cutoff value doubled the specificity, from 41% to 84%. The incidence of < 37 weeks’ spontaneous PDs among BV pregnant women with a pH= > 4.5 was 3.8%. The RR of spontaneous PD < 34 and <37 weeks among BV women with pH > =4.5, as compared with those with intermediate state, were 1.24 and 1.86, respectively (Fisher’s exact test, p value = 1; 0.52, respectively, both ns). No spontaneous case of PD or abortion was associated with severe or moderate AV.

**Conclusions:**

A high prevalence of asymptomatic BV was observed without statistically significant difference between black and white women. The RRs of spontaneous PD < 34 and <37 weeks among women with BV, as compared with those with intermediate state were not statistically significant but were consistent with those found in the literature.

## Background

Spontaneous preterm deliveries that occur before the 35th week of gestation have been strongly associated with intrauterine infection [1,2]. Approximately half of the neurological disabilities found in children, including cerebral palsy, are attributed to prematurity [1,2]. Among risk factors that could be modified, bacterial vaginosis (BV), is the most important [3].

Bacterial vaginosis (BV) is a modification of the vaginal flora characterized by a diminished or absent flora of lactobacilli, which increases the vaginal pH, and a significantly increased colonization of several anaerobic or facultative microorganisms, mainly *Gardenerella vaginalis, Prevotella sp, Bacteroides sp, Mobiluncus sp*, gram positive cocci, and genital mycoplasma (*Mycoplasma hominis* and *Ureaplasma urealyticum*). Bacterial vaginosis is, however, a condition in which the vaginal secretion presents scarce inflammatory signs, there are no parabasal cells and leukocytes are rare, in a similar way to what occurs in women whose mucosa is normal. That is why the term used to define the condition is “vaginosis” and not “vaginitis” [4].

Studies conducted with pregnant and non-pregnant women suggest that BV in the lower genital tract is associated with infection of the upper tract by BV microorganisms. Microorganisms may reach the amniotic cavity and the fetus, generally, by ascending from the vagina and bottom of the uterus, according to histological and microbiological studies. This could result in the inflammation of the decidua and chorio-amnion and cause subsequent preterm delivery as well as fetal and neonatal infection [1,2].

BV is the most prevalent vaginal disorder in adult women in the world, it is present in 8-23% of normal pregnant women in developed countries, and this proportion almost doubles in high-risk populations [5]. The prevalence varies according to gestational age, ethnicity and socioeconomic status; it also varies in different regions of the world even when a same protocol is used [6-9]. The prevalence of BV in the United States, estimated by the 2001–2004 National Health and Nutrition Examination Survey [10], for women between the ages of 14 and 49 years, was 29%, varying with age, race, education and poverty; black women presented higher odds of BV than white women, after adjustment for other socio-demographic characteristics. The same survey confirmed that most women with BV, 84%, are asymptomatic [11].

The reasons for the wide variations in the prevalence of BV are only partially known as are their consequences in terms of morbidity. More knowledge about them and a better understanding of the pathological processes that result in BV in different circumstances have been recognized as a research gap at a workshop on BV sponsored by the NIH/USA in 2008 [5]. Studies related to BV are very scanty outside the USA and Europe.

A meta-analysis to assess BV as a risk factor for preterm delivery [12] included 18 studies, involving more than 20 thousand women, most of whom examined around the 24th week, found that BV increases twice the overall risk of preterm delivery before 37 weeks. However, for those women tested before 20 weeks’ gestation, the odds ratio was higher than 4; for those tested with less than 16 weeks, the odds ratio was higher than 7. It also found that early BV, around the end of 1st trimester, increased the risk of spontaneous abortion, with an odds ratio of nearly 10. On the other hand, the selected studies that included women after 20 weeks’ gestation and assessed the risk of preterm delivery less than 34 and 32 weeks’ gestation did not find association with BV.

The Nugent method [13] is a standardized method, designed to evaluate bacterial vaginosis. It is highly associated with BV clinical signs and vaginal pH [13,14]. The relatively low sensitivity of BV tests for preterm birth, close to 30% (both Amsel’s and Nugent’s), is similar to that of other screening tests for spontaneous premature birth in asymptomatic women, such as the Bishop test, cervical ultrasound and fetal fibronectin [15,16]. A systematic review, involving more than 17 thousand asymptomatic women in the second trimester, compared the accuracy of three different tests for bacterial vaginosis_ Gram staining using Nugent’s criteria (7 studies), Gram staining using Spiegel’s criteria (2 studies) and Amsel’s clinical plus wet mount microscopy criteria (2 studies) _ to predict spontaneous preterm birth (< 37 weeks). Meta-analysis showed that there was no difference in the accuracy of those tests in predicting spontaneous preterm birth [17].

Hauth et al. [18], in a secondary analysis of two large samples of asymptomatic 8–22 weeks’ gestation women that were recruited for a BV trial, found that women with a vaginal pH = > 5.0 or = > 4.5 plus a Nugent score of 9–10 had significantly increased preterm birth rates (<37, <35 and <32 weeks’ gestation) and birthweigth less than 2500 g or less than 1500 g. Two studies have found that the so-called intermediate level (Nugent score 4–6) presents risk for premature birth close to that of BV (score 7–10) [18,19].

### Aerobic vaginitis

According to Donders et al. [20], a condition they have called aerobic vaginitis (VA), with or without the presence of concomitant bacterial vaginosis, may, theoretically, be a better candidate than BV to cause of pregnancy complications, such as ascending chorioamnionitis, preterm premature rupture of membranes and preterm delivery. They examined more than 600 pregnant and non-pregnant women’s vaginal smears, symptomatic and asymptomatic, on fresh wet mount microscopy, in an observational study in Belgium. Smears that were deficient in lactobacilli and positive for cocci or coarse bacilli, positive for parabasal epithelial cells, and/or positive for vaginal leucocytes were classified as aerobic vaginitis (AV), following criteria proposed by the authors.

Considering the existing knowledge gaps about BV, particularly in South America, the present study aimed a) to determine the vaginal pH level and the prevalence of asymptomatic BV before 20 weeks’ gestation in a low socioeconomic and multiethnic South American population; b) to describe the association of BV with aerobic vaginitis; c) to determine the accuracy of vaginal pH level to predict BV; d) to determine whether early asymptomatic BV and AV are associated with spontaneous preterm delivery before the 34th and the 37th week of gestation in that population.

## Methods

Asymptomatic pregnant women admitted before the 20th week of pregnancy in public prenatal services, in Rio de Janeiro, between 2006 and 2008, were evaluated a) to exclude women with clinical conditions associated with elective preterm delivery - like diabetes, hypertension, and low urinary infection-, with symptomatic vaginal conditions, with recent use of corticotherapy or antibiotic therapy, and with multiple gestation and cervical incompetence, and b) to identify pregnant women with previous history of preterm delivery,as part of a randomized controlled trial of probiotics in BV [21,22]. The latter (b) are not considered in the present study. Women who tested positive for syphilis, toxoplasmosis, gonorrhoea, or HIV were also excluded. Women reporting vaginal discharge were excluded only if a vaginal smear analysis showed bacterial vaginosis, trichomoniasis, or candidiasis; women with macroscopic genital lesions or microscopic pre-cancerous HPV-related lesions were excluded. Finally, women who presented previous history of preterm delivery or late abortion were not included in the present analysis.

After written informed consent was obtained, a vaginal pH assessment was performed. A cervicovaginal smear was then obtained from those women with a pH level > = 4.5; it was Gram-stained to assess the Nugent score and the presence of BV (Nugent score from 7 to 10) or an intermediate-degree infection (Nugent score from 4 to 6) [13]. Although the present paper is mostly a secondary analysis of the placebo group of a randomized trial of probiotics, smears were also taken from one hundred consecutive cases of vaginal pH < 4.5, who were not included in the trial, for comparison purposes. Moreover, subsamples of Gram-stained vaginal smears selected from intermediate-degree infection and BV women, with a 2 fold-higher probability of sampling those women presenting intermediate state (Nugent score from 4 to 6) and a focus on pH score higher than 4.5 (less than 5% presenting pH < 5.0), were further analyzed to determine the presence of aerobic vaginitis using a modified Donders’ score [20].

Women who were BV positive or intermediate state were eligible to the trial and were followed up for the events spontaneous preterm delivery (spontaneous PD), abortion and stillbirth; they were considered for the analyses of risk carried out in the present study, except for those women who were allocated into the intervention arm group and received the allocated intervention. Also, patients with late presentation of excluding conditions were not considered for the present risk analyses: 6 women due to pregnancy-induced hypertension, 1 individual because of overweight and 3 patients because of hematologic conditions. Vaginal pH and Nugent scores were also determined about 8 weeks after admission to the trial, around 24 weeks’ gestation, for those randomized women who attended the corresponding prenatal care visit.

Primary outcomes under study were spontaneous preterm delivery < 34 and <37 weeks’ gestation, stillbirth and abortion. A premature delivery was considered to be spontaneous if it started spontaneously (with a preterm premature rupture of membranes or with contractions), no matter the mode of delivery [23]. Gestational age was generally determined by ultrasound.

### Laboratory methods

– Measurement of vaginal pH

Vaginal pH was measured by a strip with discrimination of 0.5.

– Analyses of vaginal smears

A Dacron swab was used to obtain a cervicovaginal smear that was Gram-stained, examined and interpreted according to the criteria described by Nugent et al. [13]. Besides the requirement for Gram-staining the vaginal smear, the method uses only morphotypes which were found to present good inter-observer agreement (large gram-positive rods, G.vaginalis and Bacteroides spp. morphotypes), and curved gram-negative rods (Mobiluncus spp. morphotypes). The Nugent score classifies the vaginal flora according to the semi-qualitative presence of lactobacilli, *Gardenerella vaginalis* and bacteria similar to Gardenerella (such as *Prevotella, Bacteroides and Porphyromonas sp*), and *Mobiluncus:* a Nugent score from 0 to 3 indicates a normal flora; from 4 to 6 is called intermediate state, and from 7 to 10 indicates BV [13]. A complementary analysis of the vaginal smear was performed (400 × magnification, conventional microscope) consisting of the determination of the number of leukocytes per field and per epithelial cell, the presence and the proportion of parabasal epithelial cells, along with a further assessment of the vaginal flora, so to allow for the detection of moderate and severe aerobic vaginitis according to a modified Donders’ score [20], which did not consider the variable “proportion of toxic leukocytes”, as it could not be assessed after Gram-staining. A composite AV score of <3 corresponds to ‘no signs of aerobic vaginitis’, 3 – 4 to ‘light AV’, 5 to 6 to moderate AV, and any score >6 to ‘severe AV’, which corresponds to the already known entity *‘desquamative atrophic vaginitis’.* Without the toxic leukocyte criterion, the upper limit of the modified Donders’ score range was 8, instead of 10; otherwise, the criteria and the classification were maintained. All vaginal smears were analyzed by a same pathologist who was blind to the trial allocation arm, to characteristics of the pregnant woman and to outcomes.

### Statistical analyses

Two sided-p value for the Chi square and the likelihood ratio tests were calculated to analyze the association between vaginal pH level and Nugent class. Two sided-p values of the Fisher’s exact test were calculated to determine the statistical significance of relative risks (RRs) of spontaneous preterm deliveries; for all analyses, two-tailed probability values of .05 were considered statistically significant.

The study was approved by an Institutional Review Board (IFF/FIOCRUZ) and by the National Review Board (CONEP); it followed the ICH/GCP regulation. The study was registered at the NIH register platform; its identifier was NCT00303082.

## Results

Nearly 50% of the screened pregnant women were clinically eligible for the study; of these, 11.2% refused to give written informed consent for the determination of their vaginal pH level and the collection and analysis of their vaginal smears. Vaginal pH was then determined for 1,699 women. The distribution of the pH level and ethnicity is presented in Table 1: 70.9% had pH = > 4.5 while 34.4% had pH > =5.0; the mode was located at pH 4.5.The percentage of black women in this population (n = 1699) was 24.4% while 32.1% were white and 41.4%, interracial.

**Table 1:** Distribution of risk variables of clinically eligible pregnant women

**a) pH score**	**%**
3.5	1.0
4	28.1
4.5	36.5
5.0	25.0
5.5	9.0
6.0	0.4
Total (N = 1699)	100.0
**b) Ethnicity**	**%**
White	34.1
Interracial	41.5
Black	24.4
Total (N = 1699)	100.0
**c) Gestational age at smear collection (pH score > =4.5)**	**%**
< 14 wks	42.4
14-20 wks	57.6
Total (N = 1199)	100.0

### Subsample of women with vaginal pH < 4.5

The analysis of the vaginal smears of a subsample of clinically eligible women with pH level < 4.5, n = 100, showed that more than 95% of the corresponding Nugent scores were normal (0–3); the mode was Nugent score 1. None of the scores revealed a BV case.

### Subsample of women with vaginal pH > = 4.5

The Nugent score and class were then determined for 1,199 out of 1,204 asymptomatic pregnant women with a vaginal pH score > =4.5; the percentage of women (n = 1199) whose smears were collected before 14 weeks’ gestation (1st trimester) was 42.4%. They showed a bimodal distribution with score modes at Nugent score 1, within the normal range, and Nugent score 8, within the BV range. Only 16.4% of those women presented intermediate state; 39.8% presented BV (Tables 2 and 3).The percentage (n = 521) of smears classified as BV which presented Mobiluncus species was 29.6%.

**Table 2:** Prevalence of BV according to Ethnicity

**% with BV**		
	**All clinically eligible patients (N = 1699)**	**Clinically eligible patients with pH score >=4.5(N = 1199)**
White	28.1	40.6
Interracial	24.6	36.1
Black	32.5	44.6
All	28.1	

**Table 3:** Distribution of patients with vaginal pH > = 4.5 according to Nugent score and class (N = 1199)

**Nugent score**	**N (% of grand total)**	**Nugent class N (% of grand total)**
0	80 (6.7)	
1	256 (21.4)	
2	136 (11.3)	
3	52 (4.3)	
		**Normal**
		524 (43.7)
4	36 (3.0)	
5	49 (4.1)	
6	113 (9.4)	
		**Intermediate**
		198 (16.5)
7	156 (13.0)	
8	234 (19.5)	
9	60 (5.0)	
10	27 (2.3)	
		**BV**
		477 (39.8)
Total		1199 (100.0)

The overall prevalence of BV considering all clinically eligible women (n = 1699) can be estimated in 28.1% (95% CI: 26.0% -30.3%) and the intermediate state, in11.9%. The prevalence of BV among white women was 28.1% (95% CI: 24.6%-32.0%), among black women was 32.5% (95% CI: 28.2%-37.2%) and among interracial, 24.6% (95% CI: 21.5%-27.9%).

Considering women with a pH score = > 4.5 (n = 1199), the prevalence of BV among white women was 40.6%, among interracial, 36.1% and among black women was 44.6%.

Changes in Nugent class after an 8 week-follow up are shown through the cross-tabulation of the Nugent classes at admission (lines) and at the 8 week-follow up visit (columns)(Table 4) for those randomized women who had not been under the effect of the intervention and attended the corresponding prenatal care visit. 39% (95% CI:40.0-38.0) of the initially BV positive women presented scores at follow up that corresponded to either normal or intermediate state; 35.5% of those women originally in the intermediate state crossed over to BV scores. For this particular sample, which does not comprehend normal class subjects at admission, BV prevalence decreased 27.8% (95% IC: 22.6%-33.6%) within 8 weeks. The Nugent scores that presented the highest percentages of reduction were the 10 and the 9 followed by the score 8 (not shown). Modifications of vaginal pH levels were less pronounced.

**Table 4:** Changes in Nugent class after 8 weeks

**Nugent class 8 weeks after admission**				
	**Normal**	**Intermed**	**BV**	
**Nugent class at admission**				Total
Intermediate (N) (% of grand total)	22 (8.7)	18 (7.1)	22 (8.7)	62 (24.6)
BV (N) (% of grand total)	50 (19.8)	24 (9.5)	116 (46.0)	190 (75.4)
Total (% of grand total)	72 (28.6)	42 (16.7)	138 (54.8)	252 (100.0)

The cross tabulation of pH level and Nugent score for women with a pH level > =4.5 showed that of those women with pH level > =5.0, 67.0% had BV while for those with pH level = 4.5, only 14.1% presented BV and 71% were normal.

### pH and Nugent, association

The proportion of women presenting BV increased with pH levels, as expected (Table 5), and the sharpest increase (18.2 to 56.8) was observed at vaginal pH = 5.0. P values (<0.001) of both the *chi* square and the likelihood ratio tests point to a significant association between vaginal pH and Nugent class (normal, intermediate, BV).

**Table 5:** Cross tabulation of vaginal pH level and Nugent class of pregnant women, pH score > = 4.5

**pH score**	**Nugent class (N,% within class)**			
	**Normal**	**Intermediate**	**BV**	**Total N (% of grand total)**
4.5	438 (83.6)	92 (46.5)	87 (18.2)	617 (51.5)
5.0	71 (13.5)	81 (40.9)	271 (56.8)	423 (35.3)
5.5	15 (2.9)	25 (12.6)	111 (23.3)	151 (12.6)
6.0	0 (0)	0 (0)	8 (1.7)	8 (0.7)
Total	524 (100)	198 (100)	477 (100)	1199 (100)

Pearson Chi-Square, df = 6, 2-sided, p < 0.0001.

Likelihood Ratio test, df = 6, 2-sided, p < 0.0001.

### Modified Donders findings

None of the microscopic readings for women with a vaginal pH < 5.0 revealed cases of either moderate or severe AV (modified scores, excluding the criterion related to toxic leukocytes); none of the 137patients with vaginal pH > =5.0 presented severe AV and less than 5% presented moderate AV. The joint distribution of AV classes (modified Donders’ score) and Nugent classification showed that among women with Intermediate Nugent scores only 5.2% were classified as moderate AV, none as a severe case. Finally, only 3.7% of the BV positive cases were classified as moderate AV, none was classified as severe AV (modified scores). Less than 10% of the patients whose smears were analyzed presented more than 10 leukocytes hpf: they were generally associated with very low Nugent scores; none was associated to adverse events.

### pH analysis for predicting BV status

Tables 6 and 7 show BV status according to vaginal pH level, assuming 2 different cutoff values and two subsets of population: the one represented in Table 5, pH= > 4.5, and the one corresponding to pH < 4.5 (29.1%, Table 1a). For the latter subset, the Nugent scores distribution followed that obtained for the subsample with pH < 4.5, as reported above. The sensitivity of vaginal pH determination to predict BV status, considering the 4.5 cutoff value, was 100%, as expected, while for the 5.0 cutoff value it was 82%. On the other hand, the 5.0 cutoff value doubles the specificity, from 41% to 84%.

**Table 6:** Sensitivity and specificity of vaginal pH to predict BV status, cutoff value: pH = 4.5

**pH**	**BV+**	**BV-**	**Total**
< 4.5	0	492	492
**> =** 4.5	477	722	1199
**Total**	477	1214	1691

Sensitivity: 477/477 = 100%.

Specificity: 492/1214 = 41%.

Accuracy: 57.3%.

**Table 7:** Sensitivity and specificity of vaginal pH to predict BV status, cutoff value: pH 5.0

**pH**	**BV+**	**BV-**	**Total**
< 5	87	1022	1109
≥5	390	192	582
**Total**	477	1214	1691

Sensitivity: 390/477 = 82%.

Specificity: 1022/1214 = 84%.

Accuracy: 84%.

### Risk analyses: early mid trimester vaginal pH level and BV for predicting spontaneous PD

338 women eligible to the trial who did not receive the study intervention and who had not late presentation of excluding conditions were followed up for the events spontaneous preterm delivery (PD), abortion and stillbirth and are considered for the analyses of risk below.

### Observed unfavorable outcomes

Tables 8, 9 and 10 present selected maternal data related to the observed cases of spontaneous PD <34 and spontaneous PD 34–37 weeks’ gestation and abortions. The incidence of < 37 weeks’ spontaneous PDs among BV pregnant women with a pH= > 4.5 was 3.8% (95% CI: 2.0%-7.0%). No case of stillbirth occurred in the present study sample.

**Table 8:** Selected maternal data related to cases of spontaneous preterm delivery <34 weeks’ gestation

**Case**	**Vaginal pH**	**Nugent score**	**Modified**	**Birth weight (kg)**	**GA at birth (USG)**
			**Donders’ score**		
1	5.0	10	3	2.010	30w, 6d *1
2	5.5	6**	3**	1.695	33w, 2d
3	4.5	9	3	1.580	31w, 4d
4	5.0	8	3	2.305	33w, 6d *2

*1,2 The GA estimated by neonatologists were 33w 6d and 35w5d, respectively.

**Results are not reliable due to insufficient material for analysis.

**Table 9:** Selected maternal data related to cases of abortion

**Case**	**Vaginal pH**	**Nugent score**	**Modified**	**Birth weight (kg)**	**GA at birth (USG)**
			**Donders’ score**		
**Case 1**	5.0	7	2	-	12w, 3d

**Table 10:** Selected maternal data related to cases of spontaneous preterm deliveries between 34- < 37 weeks’ gestation

**Case**	**Vaginal pH**	**Nugent score**	**Modified** **Donders' score**	**Birthweight (kg)**	**GA at birth (USG)**
1	4.5	8	3	2.925	36w, 0d
2	5.5	8	4	2.555	36w, 6d
3	5.0	7	2	2.300	35w, 5d
4	5.0	8	3	2.665	36w, 6d
5	4.5	5	3	2.220	36w, 6d*
6	5.0	8	4	2.435	35w, 1d
7	5.5	7	3	2.720	35w, 5d**

*The GA based on neonatologist’s estimate was 34w, 6d.

**LMP and neonatologist’s estimate (no USG available).

It can be observed that cases of premature birth presented pH scores generally higher than 4.5. Births less than 34 weeks’ gestation presented Nugent scores between 8 and 10, except for the case with insufficient material, and modified Donders’ scores not higher than 3. Cases from 34 to 37 weeks’ gestation presented Nugent scores between 7 and 8, except for one case of Intermediate Nugent score; they had modified Donders’ scores not higher than 4.

A sensitivity analysis was performed to consider the exclusion of participants that required antibiotic therapy against vaginal or urinary infection during the follow up period until delivery (n = 52). The resulting cases of spontaneous PD less than 34 weeks’ gestation were not changed; for spontaneous PDs between 34–37 weeks, case number 7 was excluded.

### Relative risk of spontaneous PD associated with BV status

2×2 contingency Tables for categories BV positive/BV Intermediate degree infection and observed spontaneous PDs less than 34 and less than 37 weeks’ gestation, for women with a pH level > =4.5 and a Nugent score between 4 and 10, are presented in Tables 11, 12 and 13. The relative risk (RR) of spontaneous PD < 34 weeks’ gestation among BV positive women in comparison with intermediate state women (Nugent scores 4–6), with a pH > = 4.5, was 1.24 (95% CI: 0.13-11.80) and the two sided-p value (Fisher’s exact test) was 1.00, ns. The RRs of spontaneous PD 34–37 and <37 weeks’ gestation were 2.49 (95% CI: 0.30-20.43) and 1.86 (95% CI: 0.41-8.47), respectively, and the corresponding two sided-p values (Fisher’s exact test) were 0.46 and 0.52, both ns.

**Table 11:** Spontaneous Preterm deliveries <34 weeks’ gestation according to BV status of asymptomatic pregnant women with vaginal pH > =4.5

**BV status**	**< 34 wks**	**> = 34 wks**	**Total**
**BV+**	3	236	239
**BV Int**	1*	98	99
**Total**	4	334	338

*insufficient material.

RR = 1,24 (95% CI:0.13-11.80).

Two sided-p value (Fisher’s exact test) = 1.00, n.s.

**Table 12:** Spontaneous Preterm deliveries 34- < 37 weeks gestation according to BV status of asymptomatic pregnant women with vaginal pH > =4.5

**BV status**	**34-37 wks**	**Deliveries > = 37 wks**	**Total**
**BV+**	6	230	236
**BV Int**	1	97	98
**Total**	7	327	334

RR = 2.49 (95% CI:0.30-20.43).

Two sided-p value (Fisher’s exact test) = 0.46, ns.

**Table 13:** Spontaneous Preterm deliveries <37 weeks’ gestation according to BV status of asymptomatic pregnant women with vaginal pH > =4.5

**BV status**	**34-37 wks**	**Deliveries > = 37 wks**	**Total**
**BV+**	9	230	239
**BV Int**	2	97	99
**Total**	11	327	338

RR = 1,86 (95% CI:0.41-8.47).

Two sided-p value (Fisher’s exact test) = 0.52, ns.

A sensitivity analysis was performed to consider the exclusion from the analysis of 52 participants that required antibiotic therapy against vaginal or urinary infection during the follow up until delivery. The above relative risks and *p* values were not changed significantly. Another sensitivity analysis to consider as BV the spontaneous PD case related to insufficient material, instead of intermediate state (Nugent 6), regarding the outcome spontaneous PD less than 37 weeks’ gestation, resulted in a RD = 7.59/239 = 0.032, a RR = 990/239 = 4.14 and a two-sided *p* value (Fisher’s exact test) of 0.19, ns. It was not possible to calculate relative risks of spontaneous PD for categories BV positive vs BV negative (intermediate condition plus normal) women.

## Discussion

A high prevalence of BV, 28.1%, was found in public prenatal services among asymptomatic pregnant women less than 20 weeks’ gestation in the city of Rio de Janeiro. After an 8 week-follow up, nearly 40% of the initially BV-positive women turned into either normal or intermediate state. The relative risks of spontaneous PD < 34 and <37 weeks among women with BV as compared with those with intermediate state, and pH > =4.5, before 20 weeks’ gestation, were 1.24 and 1.87, respectively, both ns. Despite the non-significance, our observed RR of <37 weeks’ spontaneous PD is consistent with the odds ratio estimated in a published meta-analysis, normal smears included [12]. None of the observed spontaneous PD cases was associated with a severe or moderate case of aerobic vaginitis.

Our BV excess rate may be attributed to the fact that 42.4% of the smears were collected before 14 weeks’ gestation within the 1st trimester of gestation; the remaining, before the 20th week. The prevalence of BV is expected to decrease after the 20th week of gestation, according to longitudinal studies [24,25]. Our BV prevalence was higher than those reported by a NIH-USA study [26], 23.4%, in women less than 25 weeks’ gestation and by Hillier et al., 16% [27], at the end of the 2nd trimester of pregnancy, who considered as BV cases only those found at vaginal pH levels above 4.5; using that pH cut off, our figure would be 23%. However, our rate was lower than that found by Klebanoff et al. [28], 34.4%, in a very large sample of women between 8–22 weeks’ gestation, 39% of which were less than 13 weeks’ gestation at screening. All studies used the Nugent method.

On the other hand, our excess prevalence rates could be related to the generally low socio-economic characteristics of our sample and to psychosocial stress [29]. Also, it could be attributed to its ethnic components: 24.4% of the clinically eligible women were black and 41% were interracial. However, the prevalence of BV in black women, 32.5%, was not significantly higher than that of white women, 28.1%. These findings are in apparent disagreement with those produced by the recent systematic review carried out by Kenyon et al. [30] that observed considerable variation among ethnic groups in all continents, and with the findings of two large studies carried out in the USA [9,26]. The first study [9], of pregnant women at 23 to 26 weeks’ gestation, found a prevalence of 9% in white women whereas for black women it was 23%; after adjustment for socio-demographic and sexual behavior characteristics, the corresponding odds ratio did not change markedly. The other study [26], of pregnant women under 25 weeks’ gestation, found a prevalence of 29% in black women, nearly twice of that found in non-black women, 14%. The lack of variation according to ethnicity is consistent with the finding of another Brazilian study [31].

Our 8-week follow-up confirmed that a significant portion of the women with BV go on a spontaneous process of remission [32].

The present work also found that about 70% of those clinically eligible women had pH scores > = 4.5, a higher figure than that registered by Hauth et al. [18], nearly 60%, in a large multiethnic sample that involved pregnant women from 8–22 weeks’ gestation. About one third of those clinically eligible women had pH scores > =5.0, almost twice the frequency observed Simhan et al. [33], 18.0%, in women screened at the end of the second trimester. Those authors also found that pH levels > = 5.0 were associated with significantly higher rates of preterm delivery, particularly spontaneous deliveries and those below 32 weeks’ gestation. Only 3 out of 11spontaneous preterm deliveries observed in the present study had pH levels below 5.0; however, it was not possible to calculate the corresponding relative risk because women with pH levels below 4.5 and women with pH levels higher than 4.5 and Nugent scores within the normal range, considered as low risk subgroups, were not followed up.

In our study, the incidence of < 37 weeks’ spontaneous PDs among BV pregnant women with a pH= > 4.5 was 3.8% (95% CI: 2.0%-7.0%), a lower figure than that reported by Hauth et al. [18], Goldenberg et al. [26], and Klebanoff et al. [28], above 10%. The difference could be due to the generally less restrictive exclusion criteria adopted by those studies regarding known risk factors and elective preterm delivery. Another possible explanation could be the fact that the majority of the participants in the present study formed the placebo group of the related trial, an allocation-patient-carer-evaluator blinded experiment, and were therefore under the placebo and the Hawthorne effects. Klebanoff et al. [28] also analyzed BV data from control group participants to evaluate BV as risk factor to preterm birth as well as Leitich et al. [12] who used data relative to control group participants from 3 RCTs in their meta-analysis.

BV in early pregnancy is considered a stronger risk factor for preterm delivery, particularly under 32 weeks’ gestation, than BV later in pregnancy [12,34,35]. Although the analysis carried out by Klebanoff et al. [28] does not confirm such finding, their study sample was under 23 weeks’ gestation and 90% of their subjects were under 21 weeks at screening. The present work, whose sample was under 20 weeks’ gestation at screening, found a relative risk of spontaneous PD <34 weeks’ gestation of 1.24, not significant statistically, for BV compared to intermediate state women, at a pH score > = 4.5. The corresponding relative risk of spontaneous PD <37 weeks’ gestation was 1.86, also not significant statistically. Unfortunately, it was not possible to follow up women with normal Nugent scores, nor those with pH scores below 4.5, which could have allowed the calculation of a BV positive/BV negative RR associated with greater numbers. Our observed RRs are consistent with the corresponding odds ratios estimated in the meta-analysis by Leitich et al. [12] and with the odds ratio (OR) of preterm birth (23–36 weeks’ gestation) reported by Klebanoff et al. [28], OR =1.2, 95% CI: 1.1-1.4, whose sample had a gestational age structure at screening similar to ours.

Several studies have suggested diverse colonization processes for BV and the need to better understand the pathogenesis of BV has been pointed out [5]. A BV prevalence study of asymptomatic pregnant women by the end of 2nd trimester, in five continents, using a standardized protocol for the sample collection and interpretation (Nugent criteria) at a same centralized laboratory, found an overall prevalence of BV of 12.3%. Interestingly, none of the smears with BV from Ireland presented Mobiluncus morphotypes while in all other continents they were seen in at least 75% of the smears with BV [6]. In our study, in which all smears were analyzed by a same pathologist, Mobiluncus was observed in the smears of nearly 30% of the subjects with BV.

Donders et al. [20] suppose that aerobic vaginitis (VA), with or without the presence of concomitant bacterial vaginosis may, theoretically, be a better candidate than BV to cause pregnancy complications, such as preterm delivery. Donders et al. [20] as well as Nenadic et al. [36] claim that some patients with a mild, and the commonest, form of aerobic vaginitis (AV), are actually misdiagnosed as BV, or classified into the group with “intermediate” findings [18,19]. The present study observed that among women with intermediate Nugent scores, only 5.2% were classified as moderate AV and 3.7% of the BV positive cases were classified as moderate AV. Moreover, the observed adverse events do not seem associated with aerobic vaginitis but to BV, particularly to full BV (Tables 8, 9 and 10). Our findings do not seem consistent either with those more recently reported by Donders et al. [37] regarding the predictive value of BV and AV.

Our results cannot be, however, compared to those reported by Donders et al. if one accepts, as proposed by Donders et al. [38], that the process of Gram-staining of vaginal smears, as used in the present study, to assess lactobacillary flora, particularly the occurrence of BV, could result in loss of lactobacilli and overestimation of the occurrence of disturbances of the vaginal flora. Furthermore, stained smears make it difficult to assess the presence of toxic leukocytes and therefore the “proportion of toxic leukocytes” criterion was not considered in the modified score used in the present study, which was not validated. However, toxic change in neutrophils reflects morphologic abnormalities acquired under conditions that intensely stimulate neutrophil production, usually in response to severe inflammation, and none of the smears that presented more than 10 leukocytes hpf, expressing inflammation, was associated to severe AV or to adverse events in the present study. Another problem is that the criteria used by Donders et al. to classify smears for the presence of BV [37] are not those of Nugent et al. Those caveats do not seem, however, to explain the lack of association of our adverse events to AV findings.

## Conclusions

The prevalence of BV found in public prenatal services among asymptomatic pregnant women less than 20 weeks’ gestation in the city of Rio de Janeiro is high. The excess rate may be attributed to the fact that more than 40% of the smears were collected by the end of the 1st trimester. The prevalence of BV among black women was not significantly higher than that observed among white women. The incidence of < 37 weeks’ spontaneous PDs among BV pregnant women with a pH= > 4.5 was relatively low, 3.8%, possibly due to the study’s excluding conditions. The RRs of spontaneous PD < 34 and <37 weeks among women with BV, as compared with those with intermediate state were not statistically significant but were consistent with those found in the literature. Aerobic vaginitis was not associated with spontaneous PD.

## Competing interests

The authors declare that they have no competing interests.

## Authors’ contributions

LKS and AAH contributed with the conception of the study. LKS, AAH, MELM, KGC and AB detailed most of the operational procedures. AAH performed the microscopic analyses. LKS, MBA and AAH were involved with data analyses. All authors have participated in data collection and editing as well as in drafting the manuscript and have given final approval of the version to be published.

## Pre-publication history

The pre-publication history for this paper can be accessed here:

http://www.biomedcentral.com/1471-2393/14/107/prepub

## References

[B1] Goldenberg RL Andrews WW Hauth JC Choriodecidual infection and preterm birth Nutr Rev 2002 60 5 S19 S25 10.1301/00296640260130696 12035853

[B2] Romero R Espinoza J Chaiworapongsa T Kalache K Infections and prematurity and the role of preventive strategies Semin Neonatol 2002 7 259 274 12401296 10.1016/s1084-2756(02)90121-1

[B3] Goldenberg RL Iams JD Mercer BM Meis P Moawad A Das A Copper R Johnson F for theNational Institute of Child Health and Human Development Network of Maternal-Fetal Medicine Units What we have learned about the predictors of preterm birth Semin Perinatol 2003 27 3 185 193 10.1016/S0146-0005(03)00017-X 12889585

[B4] Cauci S Vaginal immunity in bacterial vaginosis Currt Infect Dis Rep 2004 6 6 450 456 10.1007/s11908-004-0064-8 15538982

[B5] Marrazzo JM Martin DH Watts DH Schulte J Sobel JD JD Hillier SL Deal C Fredricks DN Bacterial vaginosis: identifying research gaps. Proceedings of a Workshop Sponsored by DHHS/NIH/NIAID. Nov 19–20, 2008 Sex Transm Dis 2010 37 12 732 744 doi:10.1097/OLQ.0b013e3181fbbc95 10.1097/OLQ.0b013e3181fbbc95 21068695 PMC3137891

[B6] Tolosa JE Chaithongwongwatthana S Daly S Maw WW Gaita H Lumbiganon P Festin M Chipato T Sauvarin J Goldenberg RL Andrews WW Whitney CG The International infections in pregnancy study:variations in the prevalence of bacterial vaginosis and distribution of morphotypes in vaginal smears among pregnant women Am J Obstet Gynecol 2006 195 1198 1204 10.1016/j.ajog.2006.08.016 17074543

[B7] Purwar M Ughade S Bhagat B Agarwal V Kulkarni H Bacterial vaginosis in early pregnancy and adverse pregnancy outcome J Obstet Gynaecol Res 2010 27 4 175 181 11721727 10.1111/j.1447-0756.2001.tb01248.x

[B8] Gratacós E Figueras F Barranco M Ros R Andreu A Alonso PL Cararach V Prevalence of bacterial vaginosis and correlation of clinical to Gram stain diagnostic criteria in low risk pregnant women Eur J Epidemiol 1999 15 913 916 10.1023/A:1007673531595 10669125

[B9] Goldenberg RL Klebanoff MA Nugent R Krohn MA Hillier S Andrews WW Bacterial colonization of the vagina during pregnancy in four ethnic groups. Vaginal infections and prematurity study group Am J Obstet Gynecol 1996 174 5 1618 1621 10.1016/S0002-9378(96)70617-8 9065140

[B10] Allsworth JE Peipert JF Prevalence of bacterial vaginosis: 2001–2004 national health and nutrition examination survey data Obstet Gynecol 2007 109 2001 2004 10.1097/01.AOG.0000247627.84791.91 17197596

[B11] Koumans EH Sternberg M Bruce C Mcquillan G Kendrick J Sutton M Markowitz LE The prevalence of bacterial vaginosis in the United States, 2001–2004; associations with symptoms, sexual behaviors, and reproductive health Sex Transm Dis 2007 34 11 864 869 doi:10.1097/OLQ.0b013e318074e565 10.1097/OLQ.0b013e318074e565 17621244

[B12] Leitich H Bodner-Adler B Brunbauer M Kaider A Egarter C Husslein P Bacterial vaginosis as a risk factor for preterm delivery: a meta-analysis Am J Obstet Gynecol 2003 189 139 147 10.1067/mob.2003.339 12861153

[B13] Nugent RP Krohn MA Hillier SL Reliability of diagnosing bacterial vaginosis is improved by a standardized method of gram stain interpretation J Clin Microbiol 1991 29 2 297 301 1706728 10.1128/jcm.29.2.297-301.1991 PMC269757

[B14] Schwebke JR Hillier SL Sobel JD Mc Gregor JA Sweet RL Validity of the vaginal gram stain for the diagnosis of bacterial vaginosis Obstet Gynecol 1996 88 4 573 576 10.1016/0029-7844(96)00233-5 8841221

[B15] Meis PJ Goldenberg RL Mercer B Moawad A McNellis D Johnson F Iams JD Thom E Andrews WW The preterm prediction study: significance of vaginal infections. National institute of child health and human development maternal-fetal medicine units network Am J Obstet Gynecol 1995 173 4 1231 1235 10.1016/0002-9378(95)91360-2 7485327

[B16] Iams JD Goldenberg RL Mercer BM Moawad AH Meis PJ Das AF Caritis SN Miodovnik M Menard MK Thurnau GR Dombrowski MP Roberts JH for the National Institute of Child Health and Human Development Maternal-Fetal Medicine Units Network The preterm prediction study: can low-risk women destined for spontaneous preterm birth be identified? Am J Obstet Gynecol 2001 184 4 652 655 10.1067/mob.2001.111248 11262467

[B17] Honesta H Bachmanna LM Knoxc EM Guptaa JK Kleijnend J Khan KS The accuracy of various tests for bacterial vaginosis in predicting preterm birth: a systematic review Br J Obstet Gynaecol 2004 111 409 422 10.1111/j.1471-0528.2004.00124.x 15104603

[B18] Hauth JC Macpherson C Carey JC Klebanoff MA Hillier SL Ernest JM Leveno KJ Wapner RJ Varner M Trout W Moawad A Sibai BM for theNational Institute of Child Health and Human Development Network of Maternal-Fetal Medicine Units Early pregnancy threshold vagina pH and gram stain scores predictive of subsequent preterm birth in asymptomatic women Am J Obstet Gynecol 2003 188 3 831 835 10.1067/mob.2003.184 12634666

[B19] Cauci S Hitti J Noonan C Vaginal hydrolytic enzymes immunoglobulin A against Gardenerella vaginalis toxin and risk of early preterm birth among women in preterm labor with bacterial vaginosis or intermediate flora Am J Obstet Gynecol 2002 187 4 877 881 10.1067/mob.2002.127454 12388968

[B20] Donders GGG Vereecken A Bosmans E Dekeersmaecker A Salembier G Spitz B Definition of a type of abnormal vaginal flora that is distinct from bacterial vaginosis: aerobic vaginitis Int J Obstet Gynaecol 2002 109 34 43 10.1111/j.1471-0528.2002.00432.x 11845812

[B21] Krauss-Silva L Moreira MEL Alves MB Braga A Rebello MR Camacho KG Batista MRR Almada-Horta A Savastano C Guerra F Randomized controlled trial of probiotics for the prevention of spontaneous preterm delivery associated with intrauterine infection: study protocol Reprod Health 2010 7 14 10.1186/1742-4755-7-14 10.1186/1742-4755-7-14 20591191 PMC2911410

[B22] Krauss-Silva L Moreira MEL Alves MB Braga A Camacho KG Batista MRR Almada-Horta A Rebello MR Guerra F A randomised controlled trial of probiotics for the prevention of spontaneous preterm delivery associated with bacterial vaginosis: preliminary results Trials 2011 12 239 http://www.trialsjournal.com/content/12/1/239 10.1186/1745-6215-12-239 22059409 PMC3264514

[B23] Goldenberg R Culhane JF Iams J Romero R Epidemiology and causes of preterm birth Lancet 2008 371 75 84 10.1016/S0140-6736(08)60074-4 18177778 PMC7134569

[B24] Waters TP Denney JM Mathew L Goldenberg RL Culhane JF Longitudinal trajectory of bacterial vaginosis during pregnancy Am J Obstet Gynecol 2008 199 4 431 e1- 431.e5. doi: 0.1016/j.ajog.2008.06.061 18928996 10.1016/j.ajog.2008.06.061

[B25] Hay PE Morgan DJ Ison CA Bhide SA Romney M McKenzie P Pearson J Lamont RF Taylor-Robinson D A longitudinal study of bacterial vaginosis during pregnancy Br J Obstet Gynaecol 1994 101 12 1048 1053 10.1111/j.1471-0528.1994.tb13580.x 7826957

[B26] Goldenberg RL Lams JD Mercer BM Meis PJ Moawad AH Copper RL Das A Thorn E Johnson F Donald McNellis D Miodovnik M Van Dorsten JP Caritis SN Thurnau GR Bottoms SF The NICHD MFMUNetwork The preterm prediction study: the value of new vs standard risk factors in predicting early and all spontaneous preterm births Am J Public Health 1998 88 2 233 238 10.2105/AJPH.88.2.233 9491013 PMC1508185

[B27] Hillier SL Nugent RP Eschenbach DA Krohn MA Gibbs RS Martin DH Cotch MF Edelman R Pastorek JG 2nd Rao AV McNellis D Regan JA Carey JC Klebanoff MA for the Vaginal Infections and Prematurity Study Group Association between bacterial vaginosis and preterm delivery of a low-birth-weight infant N Engl J Med 1995 333 26 1737 1742 10.1056/NEJM199512283332604 7491137

[B28] Klebanoff MA Hillier SL Nugent RP MacPherson CA Hauth JC Carey JC Harper M Wapner RJ Trout W Moawad A Leveno KJ Miodovnik M Sibai BM Van Dorsten JP Dombrowski MP O’Sullivan MJ Varner M Langer O The Nat. Institute of Child Health and Human Development Maternal-Fetal Medicine Units Network Is bacterial vaginosis a stronger risk factor for preterm birth when it is diagnosed earlier in gestation? Am J Obstet Gynecol 2005 192 2 470 477 10.1016/j.ajog.2004.07.017 15695989

[B29] Nansel TR Riggs MA Klebanoff M The association of psychosocial stress and bacterial vaginosis in a longitudinal cohort Am J Obstet Gynecol 2006 194 2 381 386 10.1016/j.ajog.2005.07.047 16458633 PMC2367104

[B30] Kenyon C Colebunders R Crucitti T The global epidemiology of bacterial vaginosis: a systematic review Am J Obstet Gynecol 2013 doi:10.1016/j.ajog.2013.05.006. [Epub ahead of print] 10.1016/j.ajog.2013.05.006 23659989

[B31] Gondo F Silva MG Polettini J Tristao AR Peracoli JC Witkin SS Rudge MVC Vaginal flora alterations and clinical symptoms in low-risk pregnant women Gynecol Obstet Invest 2011 71 158 162 doi:10.1159/000316051 10.1159/000316051 21160139

[B32] Klebanoff MA Hauth JC MacPherson CA Carey JC Heine RP Wapner RJ Iams JD Moawad A Miodovnik M Sibai BM vanDorsten JP Dombrowski MP for the National Institute for Child Health and Development Maternal Fetal Medicine Units Network Time course of the regression of asymptomatic bacterial vaginosis in pregnancy with and without treatment Am J Obstet Gynecol 2004 190 2 363 370 10.1016/j.ajog.2003.08.020 14981375

[B33] Simhan HN Caritis NS Krohn MA Hillier SL Elevated vaginal pH and neutrophils are associated strongly with early spontaneous preterm birth Am J Obstet Gynecol 2003 189 4 1150 1154 10.1067/S0002-9378(03)00582-9 14586369

[B34] Hay PE Lamont RF Taylor-Robinson D Morgan DJ Ison C Pearson J Abnormal bacterial colonization of the genital tract and subsequent preterm delivery and late miscarriage BMJ 1994 308 295 298 10.1136/bmj.308.6924.295 8124116 PMC2539287

[B35] Kurki T Sivonen A Renkonen OV Savia E Ylikorkala O Bacterial vaginosis in early pregnancy and pregnancy outcome Obstet Gynecol 1992 80 2 173 177 1635726

[B36] Nenadic DB Pavlovic MD Cervical fluid in pregnant women: relation to vaginal wet mount findings and polymorphonuclear leukocyte counts Eur J Obstet Gynecol Reprod Biol 2006 140 165 170 18406509 10.1016/j.ejogrb.2008.02.020

[B37] Donders G Van Calsteren K Bellen G Reybrouck R Van den Bosch T Riphagen I Van Lierde S Predictive value for preterm birth of abnormal vaginal flora, bacterial vaginosis and aerobic vaginitis during the first trimester of pregnancy BJOG 2009 116 1315 1324 10.1111/j.1471-0528.2009.02237.x 19538417

[B38] Donders GG Vereecken A Dekeersmaecker A Bulck B Spitz B Wet mount microscopy reflects functional vaginal lactobacillary flora better than Gram stain J Clin Pathol 2000 53 308 313 10.1136/jcp.53.4.308 10823128 PMC1731171

